# Loss of SET reveals both the p53-dependent and the p53-independent functions in vivo

**DOI:** 10.1038/s41419-019-1484-6

**Published:** 2019-03-11

**Authors:** Ning Kon, Donglai Wang, Wei Gu

**Affiliations:** 1 Institute for Cancer Genetics, Department of Pathology and Cell Biology, and Herbert Irving Comprehensive Cancer Center, College of Physicians & Surgeons, Columbia University, 1130 Nicholas Ave, New York, NY 10032 USA; 2 National Laboratory of Medical Molecular Biology, Institute of Basic Medical Sciences, Chinese Academy of Medical Sciences, Beijing, 100005 China; 3 Department of Medical Genetics, School of Basic Medicine, Peking Union Medical College, Beijing, 100005 China

## Abstract

Our previous study showed that the oncoprotein SET acts as a new reader of unacetylated p53 for transcriptional repression. To further elucidate the physiological significance of SET in vivo, we generated *set* knockout mice. *Set* knockout mice died during embryonic development between day 11.5 and day 12.5 post coitum, exhibiting cardiac edema and open neural tube, among other developmental defects. Further analyses revealed that loss of SET leads to upregulation of p53 target genes including *p21* and *puma* without any obvious effect on p53 stability in *set* knockout embryos. Notably, the developmental defects of *set* knockout mice were significantly, but nonetheless partially, rescued by concomitant deletion of *p53*. The failure to obtain fully live *set/p53* double knockout mice suggested that p53-independent targets of SET also contribute to the embryonic lethality of *set* knockout mice. Indeed, we found that FOXO1 acts as an important target of SET and that SET-mediated regulation of FOXO1 is also acetylation-dependent. Taken together, these data underscore the importance of SET oncoprotein during embryonic development and reveal both of the p53-dependent and the p53-independent functions of SET in vivo.

## Introduction

Posttranslational modifications through ubiquitination and acetylation on p53 lysine residues have been studied extensively^1^. On one hand, p53 is kept at low levels in the absence of stresses through ubiquitination and proteasome-mediated degradation; on the other hand, p53 is acetylated to enhance p53 transcriptional activities. Interestingly, both ubiquitination and acetylation are readily detected on a cluster of lysine residues at p53 C-terminus, presenting a pair of counteracting and competing regulations that tightly control p53 activities^2–4^. To understand the precise roles of p53 C-terminus in the regulation of p53 functions, mouse models have been generated to study the potential functions of C-terminal lysine modifications. One of the mouse models, *p53-7KR* mice, replaces all seven C-terminal lysine residues of p53 with arginine residues, which eliminate both ubiquitination and acetylation altogether^5^. Surprisingly, *p53-7KR* mice showed little differences in p53 stability and transcriptional activities compared to the wild-type mice^5^ albeit some fine-tuning on p53 functions during stem cell recovery, suggesting that the absence of ubiquitination and acetylation on C-terminal lysine residues have minimal effects on p53 activities^6^. Two other mouse models were generated to address the functions of p53 C-terminus through deletion of the last exon of *p53*, which contains the seven-lysine cluster at the C-terminus^7,8^. Both studies demonstrated that deletion of the p53 C-terminus leads to elevated transcription of p53 target genes in the absence of stresses and results in premature lethality due to hematopoiesis failure^7,8^. These results are consistent with the negative regulatory functions of the p53 C-terminus, either because it contains major ubiquitination sites for p53 or the cluster of positive-charged lysine residues impede the scanning for p53 response elements by the p53 DNA binding domain. Nevertheless, these studies again are unable to pinpoint either ubiquitination or acetylation as the cause of the observed phenotypes because both modifications are lost in the deletion of p53 C-terminus. Therefore, to focus on understanding the functions of p53 C-terminal acetylation, we generated *p53-KQ* (*p53*^*KQ/KQ*^) mice, in which all seven C-terminal lysine residues are replaced with glutamine residues, since glutamine is a close resemblance of the acetylated lysine^9^.

Unlike *p53-7KR* mice, *p53-KQ* mice display severe phenotypes. The *p53-KQ* mice are born alive but die shortly due to deficiencies in brain development, seizure-like movements, and their inability to obtain maternal milk ingestion^9^. Biochemical analysis suggests that the brain abnormalities in *p53-KQ* mice are in part due to activation of p53 target genes and subsequent induction of apoptosis compared to the wild-type mice, demonstrating that mimicking acetylation of p53 C-terminus leads to elevated p53 transcriptional activities. Interestingly, *p53-KQ* mice share many similarities with the p53 C-terminal deletion mutant mice in that many of the phenotypes are caused by activation p53 target genes due to increased transcriptional activities of the mutant p53 proteins.

Subsequently, in an effort to identify acetylation specific regulation of p53, SET oncoprotein is shown to bind to the un-acetylated, but not to the acetylated, C-terminus of p53 protein. Importantly, SET also binds to p53-7KR but not to p53-KQ protein, indicating that the binding between the negative-charged acidic domain of SET and the cluster of positive-charged residues of p53 C-terminus (lysine or arginine) is critical to the interaction between p53 and SET^9^. Furthermore, our studies show that knockdown of *set* leads to increased p53 transcriptional activities, reminiscent of the phenotypes in *p53-KQ* mice, suggesting that one of the potential functions of acetylation of p53 C-terminal lysine residues is to disrupt the interaction between p53 and SET in order to activate p53 transcriptional activities.

To uncover the physiological functions of SET oncoprotein, *set* knockout mice were generated through deletion of exon 2 of *set*. The Western blot analysis showed that the SET protein was absent in *set* exon2-deleted homozygote embryos, demonstrating that deletion of exon 2 of *set* created a null allele. Notably, intercross of *set*^*+/*−^mice revealed that *set*^*−/*−^ mice died during embryonic development^9^. In the current study, we conducted a detailed analysis of the embryonic lethality of *set* knockout mice. We also attempted to rescue the embryonic lethality by crossing *set* knockout mice with *p53* knockout mice to determine if deletion of *p53* can rescue the embryonic lethality in *set* knockout mice.

## Results

### Loss of SET in mouse resulted in embryonic lethality

**Fig. 1:**
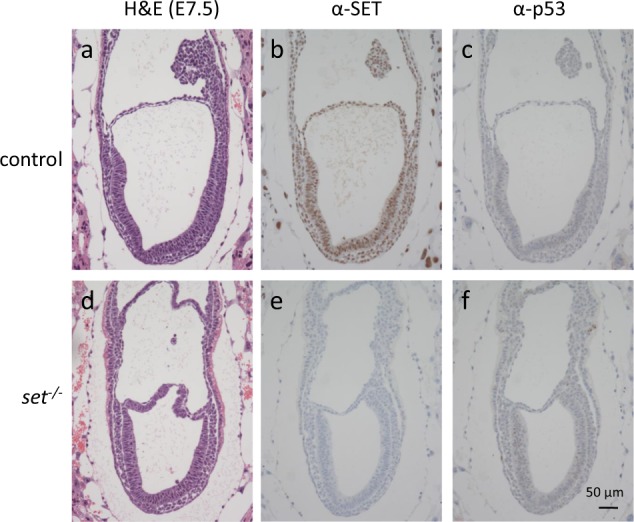
Histological analysis of E7.5 embryos from the intercross between *set*^*+/*−^ mice. **a**–**c** a control embryo; **d**–**f** *a set* knockout embryo. **a** and **d**: hematoxylin and eosin staining; **b** and **e**: immunostaining using anti-SET antibody; **c** and **f** immunostaining using anti-p53 antibody

Expanding on the previous study, *set* heterozygote knockout mice were intercrossed. From a total 52 weaning age offspring, 18 of them were wild type and 34 were *set* heterozygote knockout mice (Table 1). The ratio of wild type to *set* heterozygote knockout mice (18:34) is close to Mendelian ratio (1:2) and no *set* knockout mice have ever been recovered, consistent with the previous finding that *set* knockout mice suffered embryonic lethality. To provide a detailed analysis of *set* knockout mice during embryonic development, embryos were recovered from timed pregnancies to determine the phenotypic and biochemical changes at individual embryonic stages. We first studied embryos from embryonic day 7.5 (E7.5) of which deciduae were collected and fixed in formalin and sectioned (Fig. 1, and Supplementary Fig. 1). The sections containing the embryos were then subjected to immunostaining using an anti-SET antibody to identify *set* knockout embryos by the absence of SET staining (Fig. 1b and e). Among the total 32 embryos analyzed, 25 of them were positive for SET immunostaining, indicating that they were either wild type or *set* heterozygote knockout embryos (Fig. 1b); the other seven embryos were negative for SET staining, indicating that they were *set* knockout embryos, because deletion of exon 2 of *set* causes frame shift in protein translation and absence of SET protein (Fig. 1e). Importantly, the ratio of the combined number of wild type and *set* heterozygote embryos to the number of set knockout embryos (25:7) was close to the ratio of normal Mendelian inheritance (3:1). Moreover, there were no apparent morphological differences between SET-staining positive embryos and SET-staining negative embryos, suggesting that *set* knockout embryos developed normally during early embryonic stages up to E7.5.

**Table 1:** Summary of the intercross between *set*^*+/*−^ mice

	*set* ^*+/+*^	*set* ^*+/−*^	*set* ^*−/−*^
E7.5	25^a^		7
E8.5	5	3	4
E9.5	5	7	4
E10.5	22	42	19
E11.5	7	14	8^b^
E12.5	3	6	3^b^
Total	42	72	38
Expected	38	76	38
Weaning	18	34	0

^a^*set*^*+/+*^ or *set*^*+/−*^ embryos

^b^Dead embryos

**Fig. 2:**
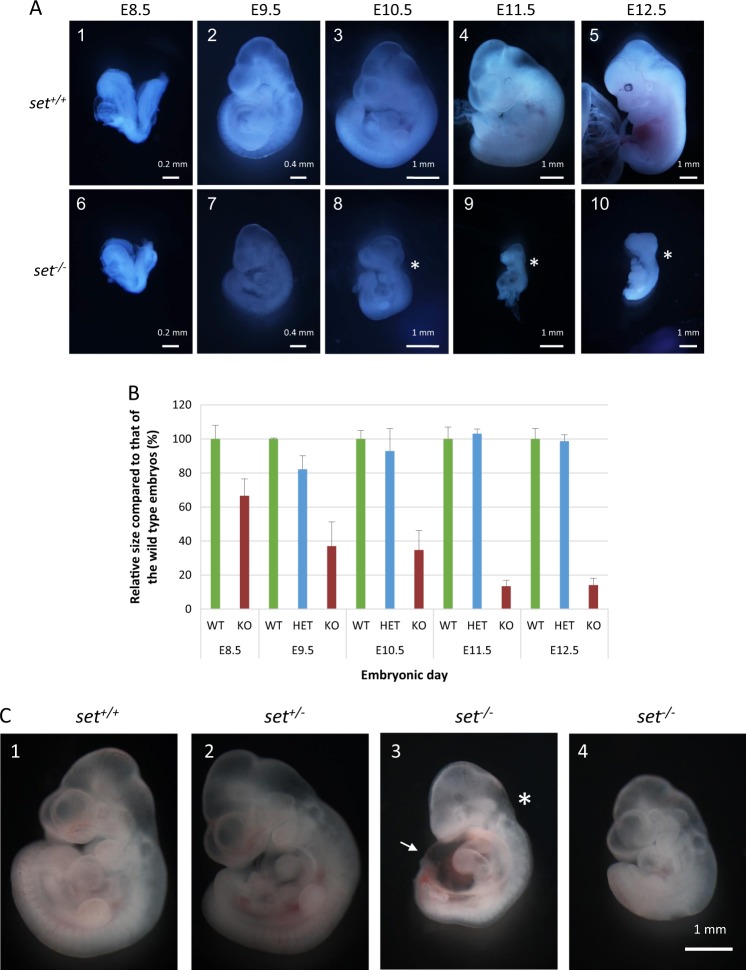
*Set* knockout mice are embryonic lethal. **a** Pictures of the representative embryos from the intercross between *set*^*+/*−^ mice are shown, 1–5: *set*^*+/+*^ embryos, and 6–10: *set*^−*/*−^ embryos. 1 and 6: embryonic day 8.5 (E8.5); 2 and 7: E9.5; 3 and 8: E10.5; 4 and 9, E11.5; 5 and 10: E12.5. **b** Comparison of the size of the embryos during embryonic development. **c** E10.5 *set*^*+/*−^ (2) is comparable to the E10.5 *set*^*+/+*^ (1) embryo. Abnormalities in the E10.5 *set*^*−/*−^ embryo include cardiac edema (3: arrow), open neural tube (3: *), and slowed growth (4)

Continuing on, we obtained later-stage embryos, of which the genotype can be determined by PCR using genomic DNA extracted from the yolk sacs. Similar to E7.5, *set* knockout embryos from E8.5 showed no significant differences compared to the wild-type embryos (Fig. 2a 6 vs. 1). However, the development of *set* knockout embryos slowed after E8.5, indicated by their smaller size compared to that of the wild-type embryos at E9.5 and E10.5 (Fig. 2a 7 vs. 2, and 8 vs. 3). The defects of *set* knockout embryos became so severe at E11.5 and E12.5 (Fig. 2a 9 vs. 4, and 10 vs. 5) that, by E12.5, all *set* knockout embryos were dead and reabsorbed (Fig. 2a 10). Significantly, the combined numbers of embryos collected from E8.5 to E12.5 were close to the Mendelian distribution of all three genotypes (Table 1). To quantify the growth defects in *set*^*−/−*^ embryos, the size of the embryos was compared using the number of pixels in the picture of each embryo. The results showed that the size of the set knockout embryos decreased gradually during embryonic development and was <20% of the size of the wild-type controls when they were close to death at E11.5 (Fig. 2b). There were no significant differences in size between the wild-type embryos and the *set* heterozygote embryos (Fig. 2b, and Fig. 2c 1 and 2). Significantly, several defects could be seen in *set* knockout embryos of E10.5, such as open neural tube (Fig. 2a 8), cardiac edema (Fig. 2c 3), and delayed growth (Fig. 2c 4), prior to their death between E11.5 and E12.5. In summary, these results confirmed that although *set* knockout mice were alive during early embryonic stages, their embryonic development was severely affected and the embryos died between embryonic day 11.5 and day 12.5, likely due to cardio vascular failure.

### p53 is activated in *set* knockout embryos

Since SET has been shown to inhibit p53 transcriptional activities through interaction with p53 C-terminus, it is important to determine if p53 transcriptional activities were changed and whether these changes caused the embryonic lethality in *set* knockout mice. For these studies, embryos of E10.5 were used because *set* knockout embryos were still alive, despite many developmental abnormalities at this stage.

**Fig. 3:**
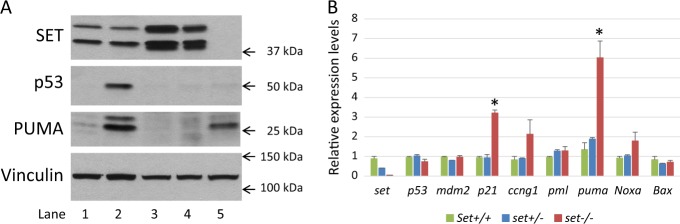
Activation of p53 target genes in *set* knockout embryos. **a** Western blot analysis using whole cell extracts prepared from spleen of wild-type control mouse (lane 1), spleen of ionizing radiation treated wild-type mouse (lane 2), E10.5 *set*^*+/+*^ (lane 3), *set*^*+/−*^ (lane 4), and *set*^*−/*−^ (lane 5) embryos, probed with the indicated antibodies. **b** Real-time PCR analysis of the relative expression levels of p53 responsive genes using total RNA prepared from E10.5 *set*^*+/+*^, *set*^*+/−*^, and *set*^*−/*−^ embryos, individually. The levels of gene expression of the *set* knockout embryos were compared to those of the *set*^*+/+*^ and *set*^+/−^ embryos. *p < 0.05

First, whole cell extracts, prepared individually from the wild-type, *set*^*+/−*^, and *set*^*−/−*^ embryos, were analyzed by Western blot probed with antibodies against SET, p53, p53 upregulated modulator of apoptosis (PUMA), and Vinculin. There was no SET protein detected in the *set*^*−/−*^ embryo, confirming the absence of SET protein resulted from the deletion of exon 2 of *set* (Fig. 3a lane 5 vs. lanes 3 and 4). As a control, a wild-type mouse was treated with ionizing radiation (IR) to induce p53 activation and protein extracts from the spleen were prepared to run along with the extracts from the embryos. As expected, p53 protein was stabilized dramatically in the mouse treated with IR, compared to that of the control mouse without IR treatment (Fig. 3a lane 2 vs. lane 1). In contrast, there were no significant changes of p53 protein levels in the *set*^−*/*−^ embryo compared to the wild-type and *set*^*+/−*^ embryos, suggesting p53 stability was not increased in the absence of SET (Fig. 3a lane 5 vs lanes 3 and 4). Notably, p53 upregulated pro-apoptotic protein, PUMA, was significantly increased in *set*^*−/−*^ embryo (Fig. 3a lane 5 vs lanes 3 and 4). PUMA was also induced in the spleen from the wild-type mouse treated with IR (Fig. 3a lane 2 vs. lane 1), confirming what was detected in the *set*^*−/−*^ embryo. Western blot of Vinculin verified similar protein levels between the extracts from these embryos (Fig. 3a lanes 3–5).

Second, to determine the effects of the loss of SET on the p53 transcriptional activities, total RNA, extracted from the wild-type, *set*^*+/−*^, and *set*^−*/*−^ E10.5 embryos using Trizol reagent, was reverse transcribed to obtain first strand cDNA, which was then used in real-time PCR (RT-PCR) to determine the relative expression levels of genes of interest (Fig. 3b). As expected, using primers in the exon 2 of *set*, the relative expression levels of *set* in *set*^*+/−*^ embryos was about 50% of that of the wild-type embryos, and there was no expression of *set* in *set*^−*/*−^ embryos. There were no significant differences in the expression levels for p53 among embryos of all three genotypes, as well as the expression levels for *mdm2*, suggesting that the transcription of *p53* was not affected (Fig. 3b). In addition, no increase in *mdm2* expression was consistent with the lack of increase in p53 abundance. Two groups of p53 responsive genes, the ones involved in cell cycle regulation and those involved in cell death, were tested. Among the genes involved in cell cycle regulation, only the expression levels of *p21* (CDKN1A) showed significant increases in *set*^*−/−*^ embryos, compared to that of the wild-type and *set*^*+/*−^ embryos (Fig. 3b). There were no significant differences for *ccng1* and *pml*. Likewise, the expression of apoptosis-associated gene *puma* increased significantly whereas no significant changes were detected for other apoptotic genes such as *noxa* and *bax* (Fig. 3b). These results showed that despite lack of changes in p53 abundance, p53 responsive genes regulating both cell cycle and cell death mechanisms were selectively activated, indicating p53 transcriptional activities were enhanced in the absence of SET. Notably, these changes in gene expression concurred with morphological and phenotypic changes of the *set* knockout embryos, as their growth stalled and the abnormalities became apparent.

Finally, embryos, collected from the intercross between *set*^*+/−*^ mice, were analyzed by immunohistochemistry to further verify the changes in p53 target gene expression. We first looked at the embryos from E7.5 as the embryos from this stage have well defined developmental features such as the emergence of the three germinal layers. There were no differences in p53 staining between the SET staining-positive embryos and SET-staining negative embryos (Fig. 1f vs. c), suggesting that the p53 abundance was not affected by the loss of SET protein in *set* knockout embryos. There were also no differences in staining for Cleaved Caspase 3, a marker for apoptosis (Supplementary Fig. 1), and p21, a gene associated with cell cycle regulation (data not shown). These results were consistent with the lack of morphological changes in E7.5 *set* knockout embryos (Fig. 1b vs a).

**Fig. 4:**
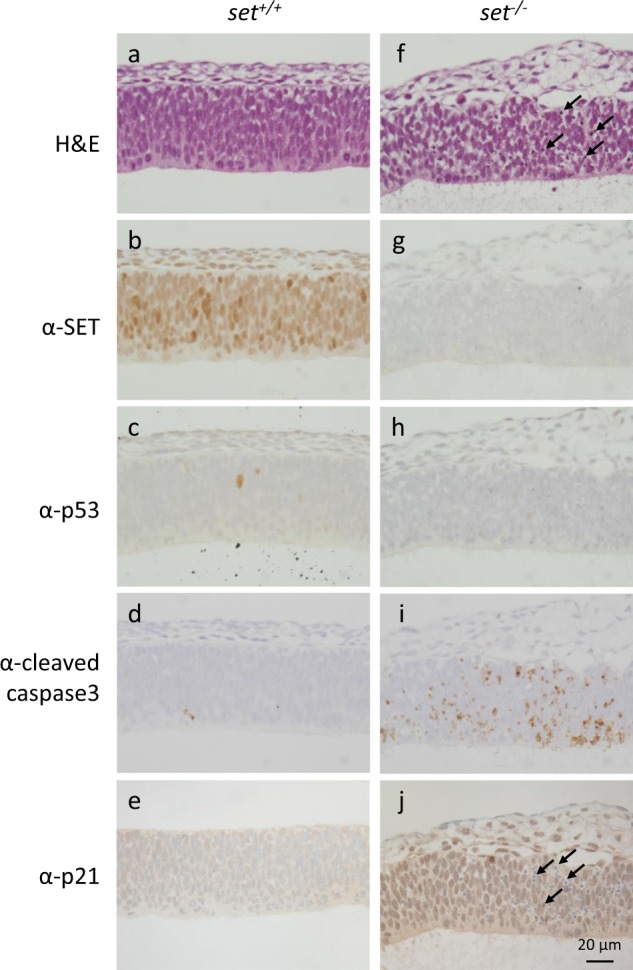
The prevalence of apoptosis and increased expression of p21 in *set* knockout embryos. Immunohistochemistry analysis of mouse E10.5 embryos. **a**–**e** *set*^*+/+*^ embryos, **f-j**: *set*^*-/-*^ embryos. **a** and **f**: hematoxylin and eosin staining; **b** and **g**: immunostaining using anti-SET antibody; **c** and **h**: immunostaining using anti-p53 antibody; **d** and **i**: immunostaining using anti-Cleaved Caspase 3 antibody; **e** and **f**: immunostaining using anti-p21 antibody. Arrows in f and j indicate pyknotic nuclei in the neuronal tissues

Similarly, the embryos from E10.5 were fixed and sagittal sections were collected. Since they are highly responsive to p53 activation, sections containing neuronal cells were analyzed in details (Fig. 4a and f, and Supplementary Fig. 2). Again, the staining for SET was negative in the *set*^−*/−*^ embryo (Fig. 4g vs. b). Notably, there were no significant differences in p53 staining between the *set*^*−/*−^ and wild-type embryos (Fig. 4h vs. c), demonstrating again that the p53 protein levels were not increased by the loss of SET in *set* knockout embryos. In contrast, there was a significant increase of staining for Cleaved Caspase 3 in the *set*^−*/−*^ embryo, compared to that of the wild-type embryo, indicating the prevalence of apoptosis in the *set*^*−/*−^ embryo (Fig. 4i vs. d, and Supplementary Fig. 2). Corroborating with the positive staining of Cleaved Caspase 3, the presence of apoptotic cells was indicated by the presence of cells with pyknotic nuclei in the brain tissues of the *set*^*−/*−^ embryo (Fig. 4f vs. a, and j vs. e). Furthermore, the immunostaining of p21 (nuclear staining), a marker for growth arrest and senescence, was elevated in almost all cells of the *set*^*−/−*^ embryo compared to that of the wild-type embryo (Fig. 4j vs. e). In summary, these results were consistent with the increased levels of transcription for *puma* and *p21*, suggesting again increased p53 transcriptional activities in the absence of SET.

### Partially rescue of the lethality of *set* knockout embryos by deletion of *p53*

**Fig. 5:**
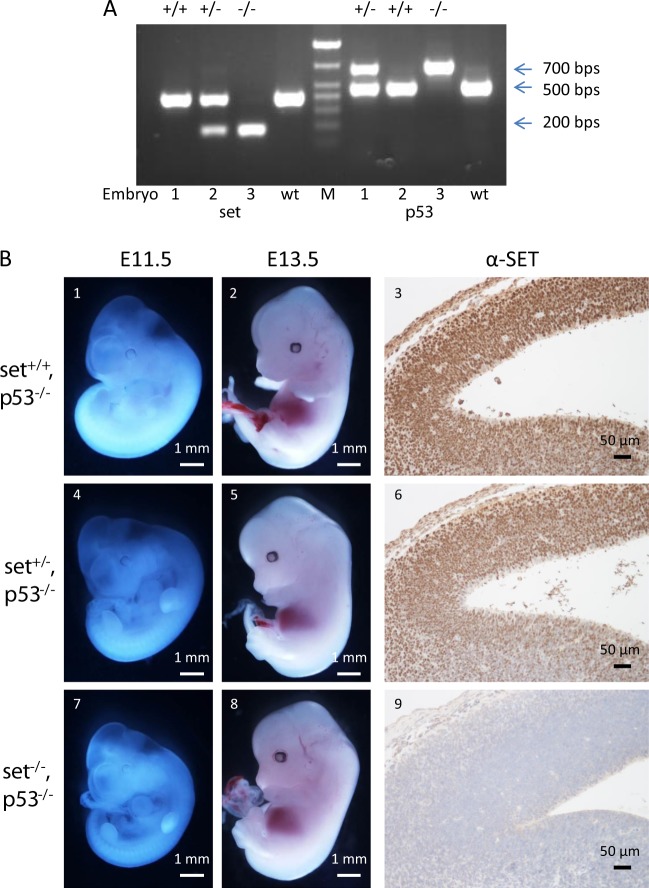
Rescue *set*^*−/−*^ embryos by crossing with *p53* knockout mice. **a** Representative genotyping results, embryo #3 is homozygote for both *set* and *p53* knockout. WT: wild type DNA control, M: 100 base-pair DNA marker. **b** Representative embryos from the cross between *set*^*+/*−^*/p53*^*+/*−^ female and *set*^*+/−*^*/p53*^*−/−*^ male mice. 1–3: *set*^*+/+*^*/p53*^*−/*−^ embryos; 4–6: *set*^*+/−*^*/p53*^−*/*−^ embryos; 7–9: *set*^−*/−*^*/p53*^−*/−*^ embryos. 1, 4 and 7: embryos from E11.5. 2, 5 and 8: embryos from E13.5. 3, 6 and 9: immunostaining of the brain sections from the embryos 2, 5 and 8, respectively, using anti-SET antibody

Because of the increased expression of p53 responsive genes in *set* knockout embryos, it was intriguing to see if deletion of *p53* could rescue the embryonic lethality of *set* knockout mice. To address this question, *set* knockout heterozygote mice were crossed with *p53* knockout mice to generate *set*^*+/−*^/*p53*^*+/−*^ female and *set*^*+/−*^/*p53*^*−/−*^ male mice, the ensuing breeding of these mice would produce *set*^*−/−*^/*p53*^*−/*−^ mice in one out of eight offspring based on Mendelian inheritance. However, we failed to identify live *set*^−*/−*^/*p53*^*−/−*^ mice after extensive breeding, suggesting that deletion of *p53* cannot completely rescue *set* knockout embryos from the embryonic lethality. We then focused our efforts to determine whether deletion of *p53* would delay the embryonic lethality in the *set* knockout embryos. Indeed, we were able to identify *set* and *p53* double knockout embryos through genotyping (Fig. 5a, embryo #3). Surprisingly, one E11.5 *set/p53* double knockout embryo was recovered with grossly normal appearance compared to the *set*^*+/+*^ or *set*^*+/*−^ embryos (Fig. 5b 7 vs. 1 and 4). However, two other *set/p53* double knockout embryos were abnormal, similar to *set* knockout embryos at E11.5 stage (Supplementary Fig. 3b, c vs. a). We then examined the embryos of E13.5 at which time point all *set* knockout embryos died. Interestingly, two out of the ten *set*^*−/−*^/*p53*^−*/−*^ embryos had normal morphologies compared to the control embryos (Fig. 5b 8 vs. 2 and 5, and Supplementary Fig. 4c vs. a and b). The loss of SET protein in the two *set*^*−/−*^/*p53*^*−/*−^ embryos with normal appearance was confirmed by the absence of immunostaining of SET in the brain sections (Fig. 5b 9 vs. 3 and 6). In addition, one *set*^*−/−*^/*p53*^*−/*−^ embryos was alive with obvious abnormalities (Supplementary Fig. 4e vs. d and f). The distribution of all possible genotypes was close to the expected Mendelian distribution, indicating no significant loss of embryos during early developmental stages (Table 2). Taken together, these data suggested that deletion of *p53* partially rescued the embryonic lethality of *set* knockout mice.

**Table 2:** Summary of the cross between *set*^*+/−*^*/p53*^*+/−*^ female and *set*^*+/*−^*/p53*^*−/−*^ male mice

E13.5	*set* ^*+/+*^	*set* ^*+/−*^	*set* ^*−/−*^
*p53* ^*+/−*^	12	26	8^a^
*p53*^*+/−*^ (expected)	11	22	11
*p53* ^*-/−*^	16	16	10 (8^a^)
*p53*^*−/*−^ (expected)	11	22	11

^a^Dead embryos

### SET regulates FOXO1 transcriptional functions through binding to unacetylated FOXO1

**Fig. 6:**
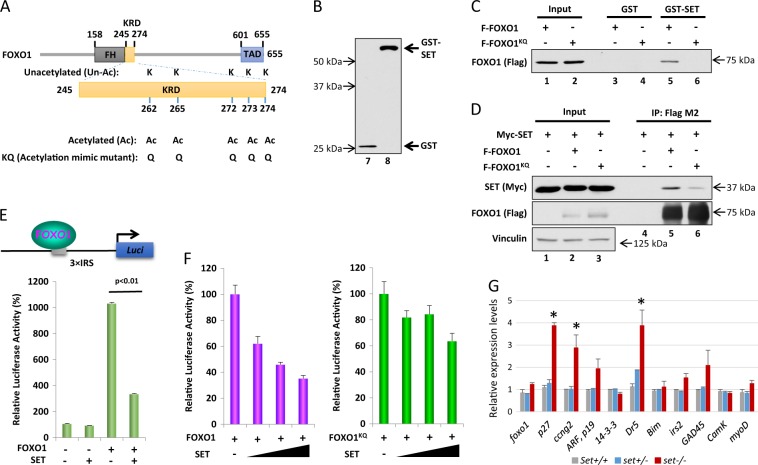
Acetylation of FOXO1 modulates its interaction with SET. **a** Schematic diagram of FOXO1. The lysine residues were substituted with glutamine to mimic FOXO1 acetylation. **b**. Western blot of purified glutathione transferase (GST) and GST-SET fusion protein. **c** In vitro binding assays (GST pull-down assay) testing the interaction between SET and FOXO1. The GST-SET interacting proteins pulled down by glutathione affinity beads were visualized by Western blot probed with anti-Flag antibody. **d** In vivo binding assays testing the interaction between Myc-tagged SET and Flag-tagged FOXO1 proteins in HEK293T cells. The interacting proteins were immunoprecipitated by M2 beads and visualized by Western blot probed with the indicated antibodies. **e** Luciferase assays of SET-mediated repression of FOXO1-dependent transcriptional activation. **f** Luciferase assays of SET-mediated repression of FOXO1- or FOXO1^KQ^-dependent transcriptional activation. Error bars indicate S.D., the samples were repeated three times for the experiment. **g** Real-time PCR analysis of the relative expression levels of FOXO1 responsive genes using total RNA prepared from E10.5 *set*^*+/+*^, *set*^*+/−*^, and *set*^−*/*−^ embryos. The levels of gene expression in the *set* knockout embryos were compared to those in the *set*^*+/+*^ and *set*^+/−^ embryos. *p < 0.05

The partial rescue of the embryonic lethality in *set* knockout mice by deletion of *p53* suggests that other genes and their regulated pathways were affected in the *set* knockout embryos. Previously, the acidic domain of SET has been shown to interact with unacetylated, but not acetylated, lysine/arginine-rich domains (KRDs) of histone H3, KU70, KLF5, and FOXO1^9–14^. Since regulation of transcription factors would have broad effects on gene expression and cellular functions, we chose to focus on the transcription factor FOXO1 to determine if the regulation by SET, through acetylation/deacetylation on lysine residues, has a functional effect on FOXO1 transcriptional activities. Similarly, we narrowed down the SET-binding domain to a lysine-arginine-rich domain in FOXO1 protein and made acetylation-mimicking mutant full-length FOXO1^KQ^ protein (K to Q mutations at K262, K265, K272, K273, and K274) (Fig. 6a). The binding specificity was first tested by an in vitro GST pull-down assay (Fig. 6b, c). The results showed that the full-length FOXO1 bound to GST-SET, in contrast, the acetylation-mimicking mutant FOXO1^KQ^ protein failed to bind to GST-SET (Fig. 6c). Moreover, the interaction between SET and FOXO1 or FOXO1^KQ^ was tested by co-expression of these proteins in HEK293T cells. As shown in Fig. 6d, the fractions of SET protein co-immunoprecipitated with FOXO1^KQ^ protein were significantly less than the fractions of SET immunoprecipitated with wild-type FOXO1. These data indicated that acetylation of FOXO1, like acetylation of p53, modulates the interaction of FOXO1 with SET. To test whether the acetylation-modulated interaction between FOXO1 and SET affects FOXO1-dependent transcriptional activation, we transfected H1299 cells with a FOXO1-reporter construct (3 × IRS-reporter) together with an expression vector for FOXO1 or FOXO1^KQ^, either with or without an expression vector for SET, to determine the FOXO1-dependent transcriptional activation by measuring the luciferase activities. Notably, FOXO1-mediated transcription activation was suppressed in the presence of SET expression (Fig. 6e). Furthermore, the suppression of FOXO1 transcription activation by SET was dosage dependent (Fig. 6f). In contrast, SET-mediated suppression of transcription was significantly abrogated when the acetylation-mimicking mutant FOXO1^KQ^ was expressed (Fig. 6f). In summary, these results suggested that FOXO1 transcriptional activities were suppressed through the interaction between SET and the unacetylated lysine/arginine domain of FOXO1. More importantly, acetylation-mimicking of the lysine/arginine domain of FOXO1 significantly relieved the transcriptional suppression by SET. To demonstrate whether such regulation has any physiological significance, the relative expression levels of FOXO1-regulated genes were determined by RT-PCR using total RNA extracted from the wild-type, *set*^*+/−*^, and *set*^−*/−*^ E10.5 embryos. The results showed that although there were no significant changes of expression of *foxo1*, the expression levels of *p27* (CDKN1B), a classical FOXO1-regulated gene, as well as those of *ccng2* and *dr5*, were significantly increased in the *set*^*−/−*^ embryos, compared to the expression levels of these genes in the wild-type and *set*^*+/−*^ embryos (Fig. 6g). There were no significant changes for many of the potentially FOXO1-regulated genes, such as CDKN2D, 14-3-3, Bim, irs2, GADD45, camK and myoD (Fig. 6g). The expression of other known FOXO1-regulated genes, such as IGFbp1, pck1, G6Pase and fas1, were below detectable levels, likely because their expression occurs at later developmental stages or their expression is linked to metabolic responses. Taken together, these results demonstrated FOXO1 as another transcription factor potentially regulated by SET through the acetylation mediated removal of the transcriptional inhibition by SET.

## Discussion

Continuing our previous study^9^, we have shown here that the p53 transcriptional activities were increased without the increase of p53 abundance in the *set* knockout mice during embryonic development. Loss of SET resulted in activation of selective p53 responsive genes, such as *p21* and *puma*, which caused growth delay and apoptosis. These findings not only provided explanations for embryonic lethality in set knockout mice but also established the inhibitory roles of SET in controlling p53 transcriptional activities without affecting p53 stability, cementing one of the functions of SET as an oncoprotein.

Many studies have shown that unchecked p53 activation in the absence of p53 inhibitors leads to embryonic lethality. However, the significance of the negative regulation of p53 by each individual inhibitor needs to be validated in vivo. As a prime example, knocking out *mdm2* in mouse results in embryonic lethality due to the increase of p53 stability and activation of p53 target genes. Fittingly, deletion of *p53* in *mdm2* knockout mice completely rescues the embryonic lethality, highlighting the importance of inhibiting p53 functions by Mdm2 under physiological conditions^15,16^. In the current study, we demonstrated significant activation of p53 target genes in *set* knockout embryos preceding their death between E11.5 and E12.5, which prompted us to determine if the embryonic lethality in *set* knockout mice can be rescued by knocking out *p53*. Notably, deletion of *p53* in *set* knockout mice resulted in significant rescue in about 20% of *set/p53* double knockout embryos as these embryos displayed relatively normal development compared to the control embryos at E13.5, at which stage all set knockout embryos died. Nevertheless, we have not obtained live *set/p53* double knockout mice at birth. The possible reasons for the inability to fully rescue *set* knockout mice by concomitant deletion of *p53* are multiple: first, we have not generated a sufficient number of mice from the breeding among which only 12.5% of all mice are *set/p53* double knockout mice. On top of that, only 20% of *set/p53* double knockout mice might survive based on the observation from the embryos at E13.5. Second, SET has been shown to affect the functions of other proteins, which are associated with many important pathways. It is possible that removing the lethal phenotypes associated with activation of p53 is not sufficient to alleviate all the damaging effects caused by the loss of SET protein. Third, our previous study of *p53-KQ* mice showed that *p53-KQ* mice are alive at birth. Even though SET protein is present in these mice, p53-KQ protein cannot interact with SET and is therefore free of the inhibitory effects imposed by SET protein^9^, suggesting activation of p53-dependent functions in the absence of SET inhibition alone cannot cause as severe phenotypes as those in the *set* knockout mice. Collectively, these results suggest that the embryonic lethality of *set* knockout embryos is the result of the combined disruption of the functions of SET-regulated proteins in the absence of SET. However, the cardiac and vascular defects seen in *set* knockout mice appear to be common abnormalities and are widely present in many mouse knockouts^17^, making it difficult to attribute any particular SET-regulated gene for the phenotypes in *set* knockout mice.

The regulation of FOXO1 by acetylation has been studied previously^18^. In this study, we verified the regulation of FOXO1 by SET, corroborated by the increased expression of FOXO1 responsive genes. Interestingly, many activated FOXO1 responsive genes have overlapping functions with those of the genes activated by p53, such as cell cycle inhibitory functions shared by p27 and p21, further suggesting that inactivating p53 in the set knockout embryos may not be sufficient to fully rescue the lethality. In addition, another SET regulated transcription factor, KLF5, is involved in heart development, potentially another hurdle needs to be overcome before *set*^*−/−*^ mice can be fully rescued.

SET oncoprotein was initially discovered due to its translocation and the resulting aberrant expression in leukemia^19–22^. The activation of p53 responsive genes in *set* knockout mice and the partial rescue of the embryonic lethality in *set/p53* double knockout mice strengthen the role of SET as an oncoprotein through inhibition of p53 transcriptional activities. Moreover, the regulation of diverse genes through charged interaction between the acidic domain of SET and the lysine/arginine-rich domain of the interacting proteins, demonstrated here for transcription factors p53 and FOXO1, suggest aberrant expression of SET would set off a ripple effect on a wide range of gene expressions due to altered transcription and changes of chromatin conformations under physiological conditions. Moreover, a recent report has shown that truncation mutations in *set* in human are associated with nonsyndromic intellectual disability, even though four out of five of those mutations in *set* present as a heterozygote in the patients, suggesting reduced expression of SET can also cause disruption of SET dependent regulation and defects in brain development and functions^23^. Therefore, better understanding of the defects in relation to SET regulated proteins could potentially lead to the development of novel therapeutic remedies such as targeting acetylation/deacetylation to circumvent the dysregulation by SET in cancer and mental illness.

## Materials and methods

### Generating *set* knockout mice

*Set* conditional knockout mice were described previously^9^. To generate *set* conventional knockout mice, the *set* conditional knockout mice were crossed with *Rosa26-cre* mice, to delete the exon 2 of *set*. The *set* heterozygote knockout mice (*set*^*+/−*^) were fertile and had no obvious abnormalities compared to the wild-type littermates. The genotyping was accomplished by PCR using primers WT 5′ (5′-TTGTGAATGGTAGGGTAATGAGA-3′) and KO 5′ (5′-GCTCGACTAGAGCTTGCGGAA-3′) with WT 3′ (5′-GAAAGAGCACCCTACCTACAGTA-3′). *p53* knockout mice were purchased from the Jackson Laboratory. Maintenance and handling protocols were approved by the Institutional Animal Care and Use Committee (IACUC) of Columbia University.

### Embryo collection and histology

Embryos were collected from timed pregnancy at various stages of gestation for comparison. The size of the embryos was determined based on the number of pixels in the embryos calculated using software ImageJ. For histological analysis, embryos were fixed in formalin overnight. Serial 5-µm sagittal sections were collected and hematoxylin and eosin staining was performed according to standard procedures. The sections were also immunostained using antibodies against SET (bethyl, A302-261A), p53 (Leica, CM5), and Cleaved Caspase 3 (Cell Signaling, 9661), p21 (Santa Cruz, SC397), and counter-stained with hematoxylin.

### Western blot analysis of the protein extracts from the embryos

The total proteins were extracted from the embryos individually using RIPA buffer. The Western blot was probed with antibodies against SET (Santa Cruz, F-9), p53 (Leica, CM5), PUMA (Santa Cruz H-136), and Vinculin. Levels of Vinculin were used as an internal protein loading control.

### Real-time PCR (RT-PCR)

The expression levels of the genes of interest were determined by RT-PCR. Briefly, total RNA was extracted from the wild-type and *set*^*−/−*^ embryos collected at E10.5 using Trizol reagent, which was then converted to first strand cDNA by reverse transcriptase. The relative expression levels of the genes were then determined by RT-PCR using the first strand cDNAs and gene-specific primers. Primers used in qPCR are: *set* forward 5′-GAACAGCAAGAAGCAATTGAAC-3′, *set* reverse 5′-TTCCTCACTGGCTTGTTCATT-3′, *p53* FORWARD 5′-GCAACTATGGCTTCCACCTG-3′, *p53* reverse 5′-TTATTGAGG-GGAGGAGAGTACG-5′, *mdm2* forward 5′-GACTCGGAAGATTACAGCCTGA-3′, *mdm2* reverse 5′-TGTCTGATAGACTGTGACCCG-3′, *p21* forward 5′-AGATCCACAGCGATATCCAGAC-3′, *p21* reverse 5′-ACCGAAGAGACAACGGCACACT-3′, *ccng1* forward 5′-CGTGTCCTCAGTTCTTTGGCTTTGACACG-3′, *ccng1* reverse 5′-GATGCTTCGCCTGTACCTTCATT-3′, *pml* forward 5′-CCAGCGTCCTGCCACAGT-3′, *pml* reverse 5′-GGTGCGATATGCATTCAGTAACTC-3′, *noxa* forward 5′-TCGCAAAAGAGCAGGAT-GAG-3′, *noxa* reverse 5′-CACTTTGTCTCCAATCCTCCG-3, *puma* forward 5′-ACGACCTCAACGCGCAGTACG-3′, *puma* reverse 5′-GAGGAGTCCCATGAAGAGATTG-3′, *bax* forward 5′-CAGGATGCGTCCACCAAGAA-3′, *bax* reverse 5′-AGTCCGTGTCCACGTCAGCA-3′, *foxo1* forward 5′-CTTCAAGGATAAGGGCGACA-3′, *foxo1* reverse 5′-GACAGATTGTGGCGAATTGA-3′, *p27* forward 5′-GACAATCAGGCTGGGTTAGC-3′, *p27* reverse 5′-TTCTGTTGGCCCTTTTGTTT-3′, *ccng2* 5′-GCTTTGACGGAAGTGAAAGTG-3′, *ccng2* reverse 5′-GAAGGTGCACTCCTGATCG-3′, *14-3-3* forward 5′-GTGTGTGCGACACCGTACT-3′, *14-3-3* reverse 5′-CTCGGCTAGGTAGCGGTAG-3′, *CDKN2D* forward 5′-GCTGCCTCTCTCGGTGAC-3′, *CDKN2D* reverse 5′-GGCCAACTCTATGATCATTTGC-3′, *DR5* forward 5′-CGGGCAGATCACTACACCC-3′, *DR5* reverse 5′-TGTTACTGGAACAAAGACAGCC-3′, *Bim* forward 5′-GGAGACGAGTTCAACGAAACTT-3′, *Bim* reverse 5′-AACAGTTGTAAGATAACCATTTGAGG-3′, *irs2* forward 5′- TCCAGGCACTGGAGCTTT-3′, *irs2* reverse 5′-GGCTGGTAGCGCTTCACT-3′, *Gadd45a* forward 5′-CCGAAAGGATGGACACGGTG-3′, *Gadd45a* reverse 5′-TTATCGGGGTCTACGTTGAGC-3′, *camK* forward 5′-TCCTTATTGGGACGACATCTCT −3′, *camK* reverse 5′-TCTTCTCCGGGTCTTTCTCC-3′, *myoD* forward 5′-CGGTCAGCCTCACAGGTC-3′, *myoD* reverse 5′-GGATGTTACAGAGCGTCAGGA-3′, and *β-actin* forward 5′-GGCTGTATTCCCCTCCATCG-3′, *β-actin* reverse 5′-CCAGTTGGTAACAATGCCATGT-3′.

### GST pull-down assay

In vitro binding assay was performed by incubating immobilized GST or GST-SET with purified Flag-tagged full-length wild type FOXO1 or Flag-tagged acetylation-mimicking mutant FOXO1^KQ^ proteins. The glutathione-agarose bound proteins were visualized by Western blot probed with anti-Flag antibody.

### In vivo binding assay

HEK293T cells were co-transfected with the expression constructs of Myc-tagged SET and Flag-tagged FOXO1 or Flag-tagged acetylation-mimicking mutant FOXO1^KQ^. The whole cell extracts were prepared and used in immunoprecipitation assays using Flag (M2) affinity beads. The M2-beads bound proteins were visualized by Western blot probed with antibodies against the Myc tag and the Flag tag. Levels of vinculin were used as an internal protein loading control.

### Luciferase assay

A Firefly reporter construct containing synthesized FOXO1-binding element (*3* *×* *IRS*-Luci) and a control Renilla reporter construct was co-transfected with the expression constructs of SET and FOXO1 into H1299 cells. The relative luciferase activity was determined based on the dual-luciferase assay protocol. All FOXO1 constructs used in the luciferase assay contain AKT phosphorylation-deficient mutations in order to maintain FOXO1 in an active form.

### Statistical analysis

Results are shown as means +/− S.D. Statistical significance was determined using a two-tailed, unpaired Student *t*-test or one-way ANOVA analysis.

## Supplementary information

Supplemental Figures

supplementary figure legends
