# Supplementary material for: Loss of SET reveals both the p53-dependent and the p53-independent functions in vivo
Source: Cell Death Dis. 2019 Mar 11;10(3):237. doi: 10.1038/s41419-019-1484-6 (PMC6411979; doi:10.1038/s41419-019-1484-6)

Supplementary Fig. 1

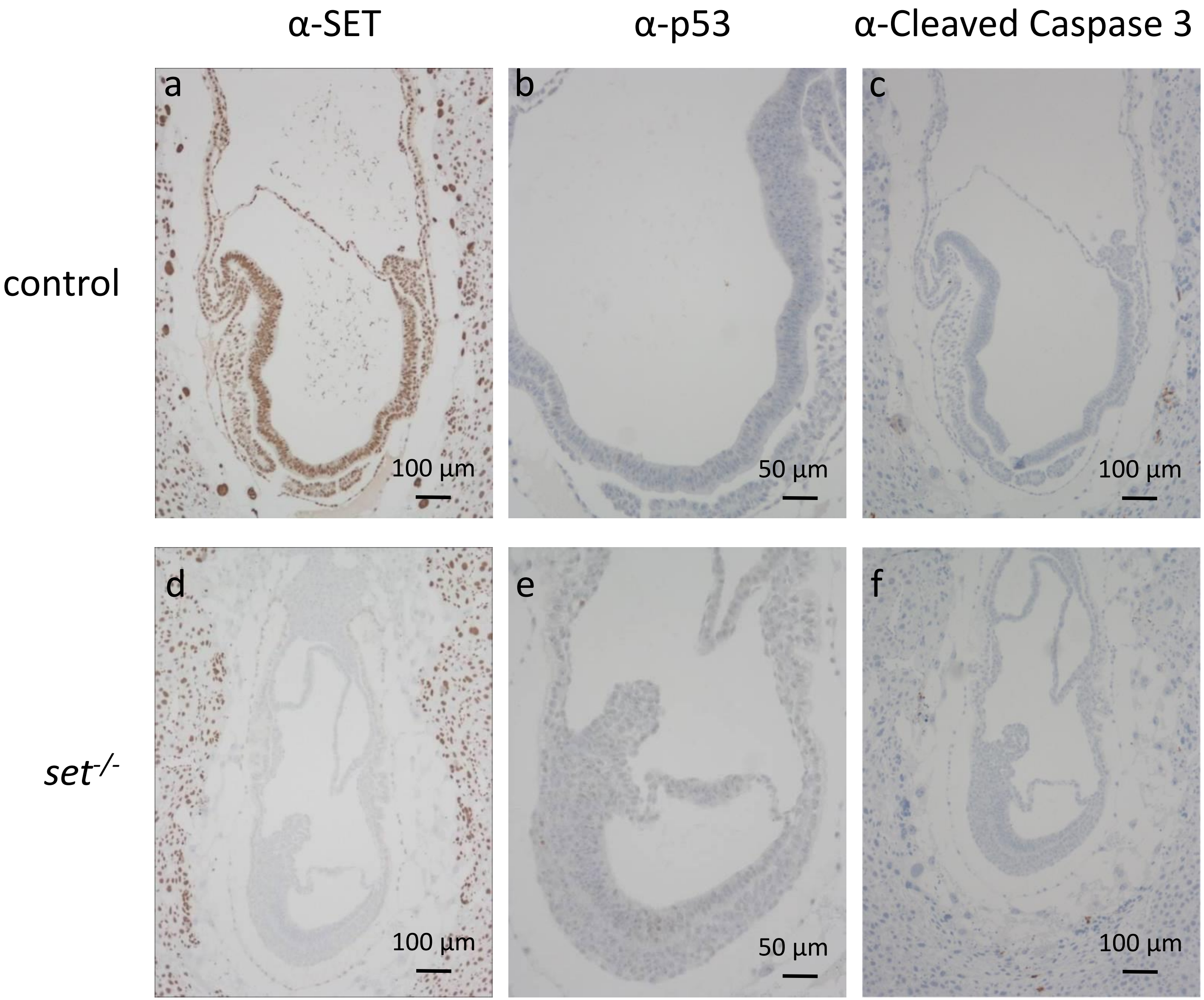

Supplementary Fig. 2

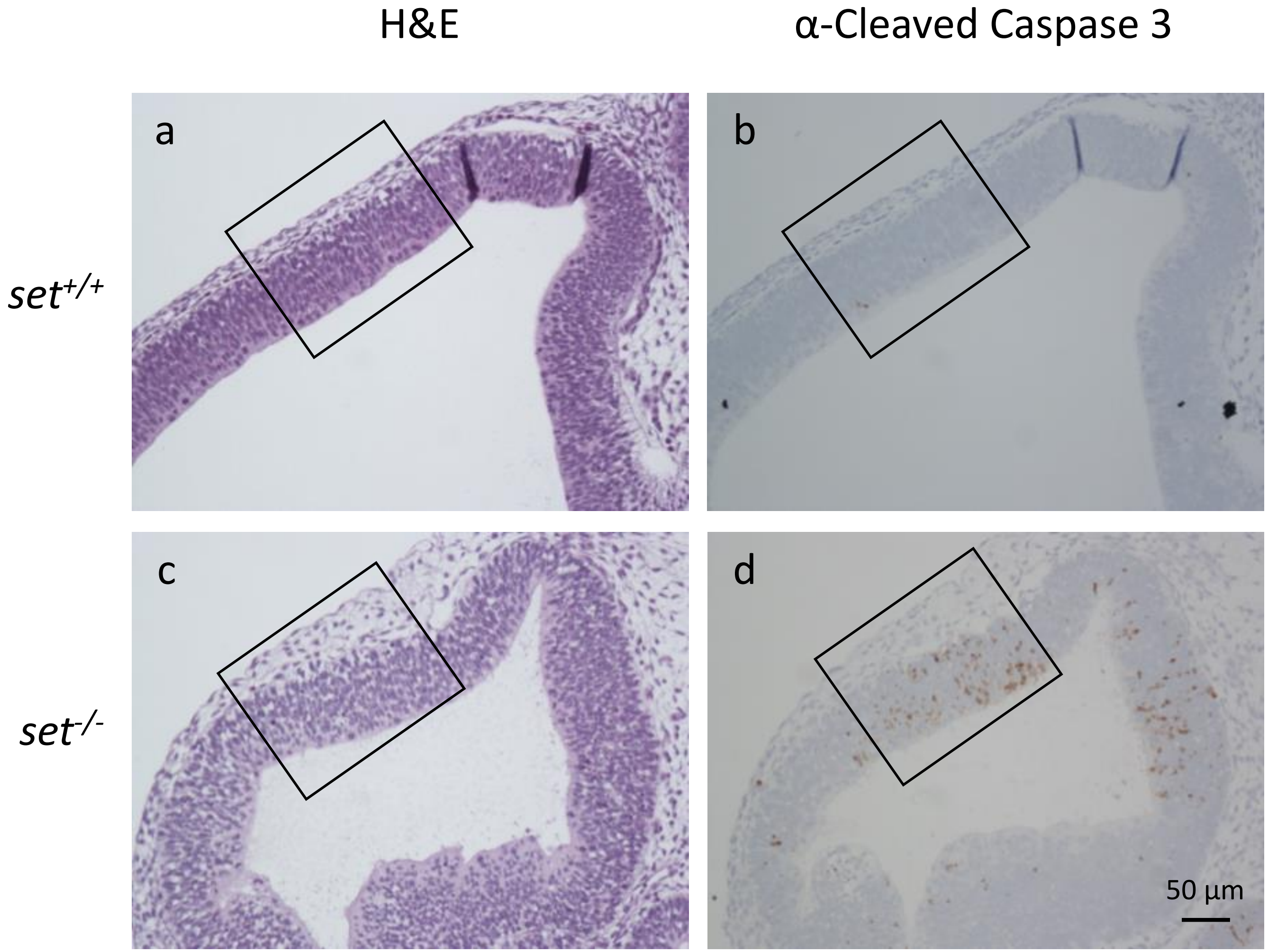

Supplementary Fig. 3

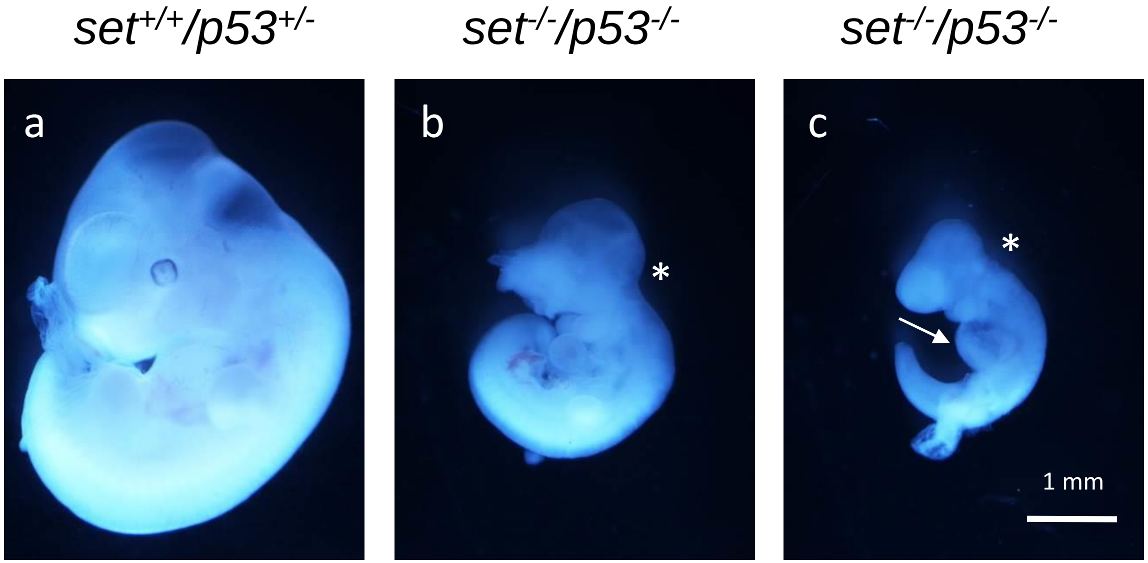

Supplementary Fig. 4

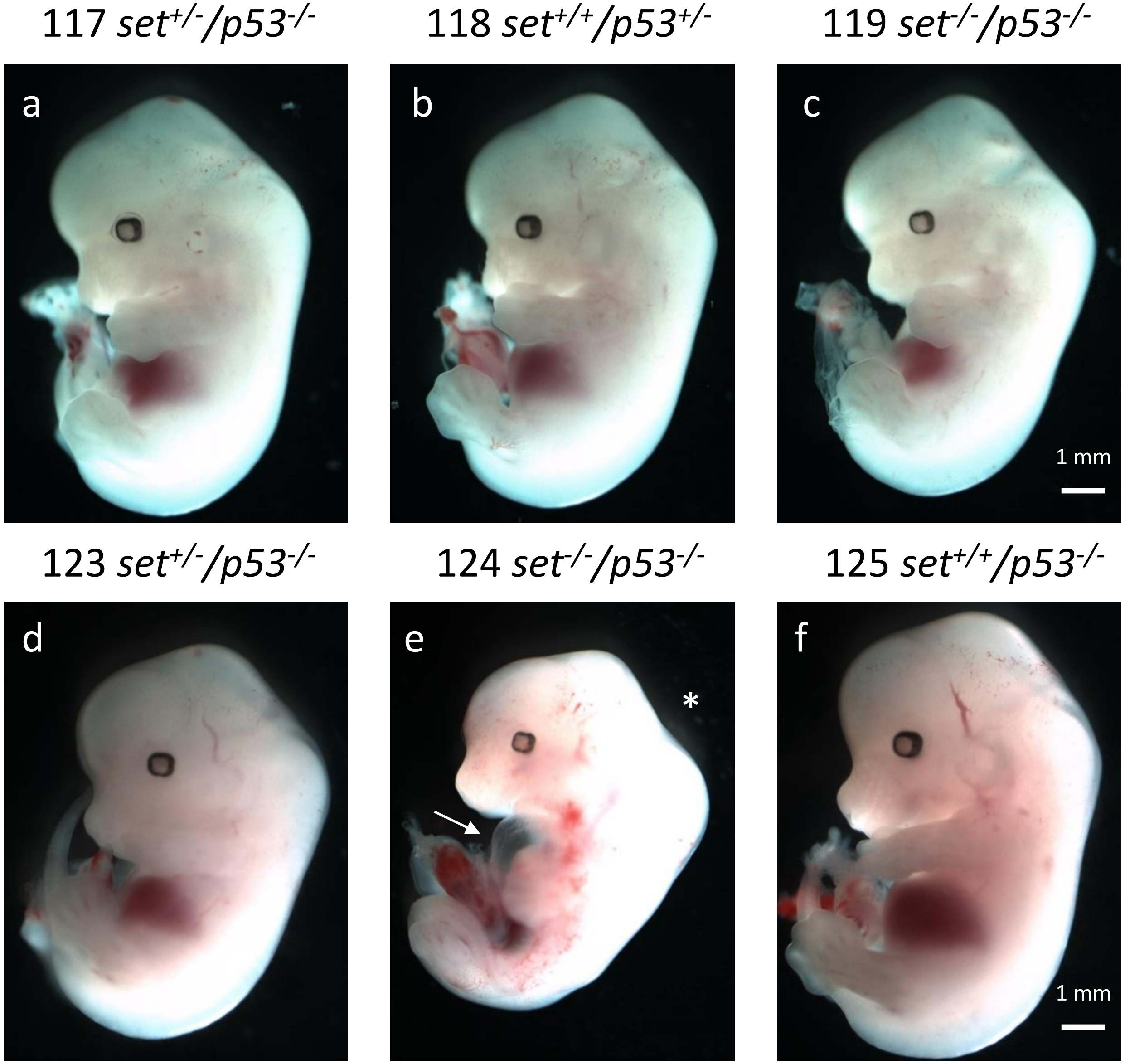

Supplement: Supplementary file 1 — Supplemental Figures [file 41419_2019_1484_MOESM1_ESM.pdf]
