# Supplementary material for: Loss of SET reveals both the p53-dependent and the p53-independent functions in vivo
Source: Cell Death Dis. 2019 Mar 11;10(3):237. doi: 10.1038/s41419-019-1484-6 (PMC6411979; doi:10.1038/s41419-019-1484-6)
Supplement: Supplementary file 2 — supplementary figure legends [file 41419_2019_1484_MOESM2_ESM.doc]

**Supplemental figure legends:**

**Supplemental fig. 1: Immunostaining of E7.5 embryos from the intercross between *set^+/-^* mice.** a-c: E7.5 control embryo; d-f: E7.5 *set^-/-^* embryo. Immunostaining using antibodies against SET (a and d), p53 (b and e), and Cleaved Caspase 3 (c and f).

**Supplemental fig. 2: Regions containing neuronal cells in E10.5 *set^+/+^* and *set^-/-^* embryos.** a and c: hematoxylin & eosin staining of regions containing neuronal cells in E10.5 wild type and *set^-/-^* embryos, which were analyzed in details in Figure 4 (rectangle boxes). b and d: immunostaining using an anti-Cleaved Caspase 3 antibody, rectangle boxes represent areas analyzed in details in Figure 4 (Figure 4 d and i, respectively).

**Supplemental fig. 3: E11.5 embryos collected from the cross between *set^+/-^/p53^+/-^* female and *set^+/-^/p53^-/-^* male mice.** a, a control embryo; b and c, two *set/p53* double knockout embryos, *: indicates open neural tube. Arrow indicates cardiac edema.

**Supplemental fig. 4: E13.5 embryos collected from the cross between *set^+/-^/p53^+/-^* female and *set^+/-^/p53^-/-^* male mice.** Representative embryos collected in the rescue cross. *Set/p53* double knockout embryo #119 (c) showed relatively normal development, compared to the control embryos #117 and #118 from the same pregnancy; whereas *set/p53* double knockout embryo #124 displayed cardia edema (arrow) and abnormal brain development (*). Embryos #123 and #125 are the embryos from the same pregnancy as #124.
